# Using rumination time to manage health and reproduction in dairy cattle: a review

**DOI:** 10.1080/01652176.2021.1987581

**Published:** 2021-10-21

**Authors:** S. Paudyal

**Affiliations:** Department of Animal Science, Texas A&M University, College Station, TX, USA

**Keywords:** Cow, cattle, rumination, monitoring, health, reproduction

## Abstract

Early detection of disease is the key to successful management of the dairy cattle which leads to timely treatment and prevention of costs associated with prolonged treatment and reduced milk yield. Electronic systems that allow for monitoring of physiological parameters like rumination, are now commercially available. This review paper discusses different aspects of rumination time that could be used to monitor the health and reproduction of dairy cattle. This review paper explored different areas where rumination time could be utilized in monitoring dairy cattle at calving, during the estrus period, during heat stressed conditions, and to detect diseases and transition cow disorders. In conclusion, rumination time could be used as an indicator of the health status in dairy cattle.

## 1 Introduction

### 1.1 Rumination time

Rumination has been defined as ‘the process of regurgitation of fibrous ingesta from the rumen to mouth, remastication, and reinsalivation followed by swallowing and returning of the material back to rumen’ (Welch et al. 1970). Rumination is an important component of the digestion process of ruminant animals with its primary role being the physical breakdown of roughages to facilitate its passage from the rumen into the small intestines (Sjaastad et al. 2003). The phenomenon of ‘chewing the cud’ or rechewing the previously ingested rumen contents is considered to be a unique feature of ruminants (Ruckebusch 1993).

Rumination is induced because of mechanical stimulation of nerve endings by the coarse and ridged feed particles in the region of the esophageal opening. The re-mastication activity during rumination reduces particle size and enables the particles to pass on to the reticulo-omasal orifice. The passage is also affected by particle shape, density, and digestibility which are altered during the rumination process (Sjaastad et al. 2003). The chewing activity during rumination stimulates the secretion of saliva, which facilitates swallowing by providing lubrication and possesses high concentrations of bicarbonate and phosphate buffers that helps in maintaining the ruminal pH at a fixed level (5.5–6.5) which is suitable for rumen microbial activity (Ruckebusch 1993). Regurgitation exposes animals to a reticular contraction, which along with the relaxation of the distal esophageal sphincter, allows a bolus of ingesta to enter the esophagus that is carried into the mouth by reverse peristalsis. The fluid in the bolus is squeezed out with the tongue, remasticated, and then swallowed again (Ruckebusch 1993).

During the process of digestion in the rumen, the uppermost layer of the rumen contains gas produced due to microbial fermentation of the ingesta (Sjaastad et al. 2003). Below the gas layer occurs a stratification of feed particles according to their difference in density; the uppermost layer being partially degraded long fibrous materials floating on top of more fluid layers that create a ‘mat’ layer. Dry, fibrous particles that enter rumen float due to buoyancy and become entangled in the floating fibrous mat in the rumen (Owens and Basalan 2016). As fermentation proceeds, the organic matter which serves as fermentation substrates gets depleted. Thus, the particles become small and dense enough to sink through the rumen mat to ventral parts of rumen. Larger particles are found to sink more slowly than smaller particles with the same density. Contractions of the reticulum and rumen help in the mixing of fore stomach contents and a transfer of particles to the omasum. The contraction also helps in regurgitation and aids in eructation of gases. The water content of digesta is absorbed in the omasum prior to its transfer to the abomasum where further digestion takes place due to enzymatic action. The digesta regurgitated is largely derived from the contents that were in the cavity of the relaxed reticulum. Opening of the cardia during regurgitation and its closure at the end of swallowing depend upon action of the same but quantitatively different, esophageal muscle layers (Ruckebusch 1993). When returned to the rumino-reticulum, the ruminated digesta does not immediately pass to the omasum; but is deposited in the dorsal part of the cranial sac of the rumen.

### 1.2 Measuring rumination time

Rumination is typically monitored through visual observation of individual animals (Schirmann et al. 2009). However, visual observation is labor intensive, time consuming, with only a small number of cows monitored at a time, and with limited accuracy (Kononoff et al. 2002; Schirmann et al. 2009; Carraway et al. 2013). Automating the monitoring of rumination is beneficial because it removes the influence of observers and may reduce the cost of obtaining information (Kononoff et al. 2002; Schirmann et al. 2009).

Indirect methods of monitoring rumination are based on jaw motion detecting devices that utilize strain or pressure gauges attached to or built in a halter (Kononoff et al. 2002; Schirmann et al. 2009; Braun et al. 2013). Braun et al. (2013) evaluated the rumination behavior using a noseband pressure sensor and observed a significant correlation between visual observation and results of the noseband pressure sensor. Bikker et al. (2014) evaluated an ear-mounted movement sensor and recommends this device to be used for rumination monitoring. Buchel and Sundrum (2014) assessed the jaw movement-based monitoring system and observed a satisfactory agreement of the results with visual observation. These devices provided useful information but the equipment had several limitations and was cumbersome. Most devices required full head halters that include moveable devices located under the jaw. These devices may be uncomfortable for the animals and may have affected their eating or rumination behavior, but numerous studies have shown that they were effective in differentiating jaw movements associated with chewing and ruminating behavior (Schirmann et al. 2009). Earlier versions of these devices used cables to connect to a computer and hence had limited utility on cows housed in tie stalls (Beauchemin et al. 1989). Memory capacities of these devices for data storage were limited and furthermore, the halter was needed to be removed to retrieve the data for download to a computer. These challenges limited the collection of continuous rumination data more than 21 days from free stall housed cows (Schirmann et al. 2009).

The recently developed rumination monitoring tags (SCR Engineers Ltd., Netanya, Israel) provide output data for rumination time. The system consists of rumination loggers, stationary or mobile readers and software for processing the electric records (Data flow software, SCR Engineers Ltd.). The logger is positioned on the left side of the neck by a neck collar (Schirmann et al. 2009). The regurgitation and rumination produce distinctive sounds that are recorded by a microphone and then it is processed and digitally recorded. The calculated data are summarized in 2-h intervals and stored in the memory of the logger for up to 22 h. The data are downloaded via readers positioned at locations within the barn (Schirmann et al. 2009). The beginning of a rumination event as defined by the software occurs when the system detects the sound associated with regurgitation. The algorithm considers rumination events to be separate if at least 30 s separate successive regurgitations (Buchel 2013). The only drawback of this system is if some problem prevents the data from being downloaded, the results are lost and overwritten (Schirmann et al. 2009).

Another similar system using an accelerometer (CowManager SensOoR®, Agis, Harmelen, the Netherlands) measures rumination based on the movement of ear during the rumination period. The CowManager SensOor^®^ records ear movement via a three-dimensional accelerometer located in the ear tag. The tag is positioned in the middle of the cow’s left ear. This sensor records rumination, feeding behavior and activity. On average, the tag is 5.1 cm long, 6.4 cm wide and 1.9 cm thick, weighing about 28 g (Reynolds et al. 2019). Data from the sensor are collected through a router stored at the local computer. Pereira et al. (2018) observed that the system can be useful even in the grazing dairy herds. Hill et al. (2017) evaluated this system in 6-wk-old calves and identified this as a valuable tool but ear placement and environmental conditions are critical for success.

### 1.3 Duration of rumination and the rumination pattern

Different authors have reported a range of rumination times (RTs) and patterns in dairy cattle. Welch (1970) reported a basic circadian pattern in rumination with cattle normally spending 8–9 h per day ruminating. However, the circadian pattern was found to be altered due to feeding frequency, feeding time, and ration composition (Lindgren 2009). Rumination activity primarily occurs at night and during resting periods in the afternoon (Lindgren 2009). Cattle spend 25–80 min ruminating per kg of roughage consumed (Sjaastad et al. 2003), and healthy matured dairy cows ruminated 7–8 h per day (Adin et al. 2009). An average RT in dairy cows without disease and stress was estimated to be 463 min/d in primiparous and 522 min/d in pluriparous cows (Soriani et al. 2012).

As reported by Lindgren (2009), most cattle ruminate about ½ to 1 h for 10–17 periods per day and during each period of rumination, they produce 30–60 boluses. Each cycle lasts for approximately 40 s and contains 30–60 chewings with a minor variation in the number of chewings per minute. A rest period of 4–8 s occurs between the two boluses during which there is no chewing.

Rumination is found to be voluntarily controlled by the animal, and the animal will stop to ruminate if it is disturbed, e.g. during milking process (Lindgren 2009). Any events that result in pain, hunger, maternal anxiety or illness also cause a drop in rumination time.

Cows can ruminate while standing, but preferably ruminate lying down and commonly lie laterally on the left side to optimize the positioning of the rumen (Albright 1993; Acatincai et al. 2010). Considering the entire time spent ruminating, cows ruminate when lying down 63.4% of the time and only 36.5% of the time in standing position. However, these patterns can be altered by environmental conditions, and, during summer, cows ruminate in the standing position more often (56% of the time; Acatincai et al. 2010). Sjostrom et al. (2019) evaluated the difference in rumination according to housing types and observed that daily rumination was greater for cows housed outdoors (509 min/d for indoor housed cows and 530 min/d for the outdoor cows).

A breed difference in rumination time has been reported (Aikman et al. 2008; Prendiville et al. 2010; Pereira and Heins 2019). Among a total of 108 animals grazing on grass, Holstein cows spent more time ruminating and had more mastications during rumination than Jersey cows. However, when expressed per unit of body weight, RT was greater for Jersey cows and they had more ruminating mastications compared with Holstein cows (Prendiville et al. 2010). Aikman (2008) also reported that Holsteins spent more time ruminating per day compared with Jerseys but when considering per unit of ingested feed, Jerseys spent more time eating and ruminating. In a recent study conducted for longer duration (4 years), Holstein cows had greater rumination than cross bred animals in the study and authors attributed this to the difference in body size of these animals (Pereira and Heins 2019). However, Gregorini et al. (2012) studied three hundred and twenty lactating dairy cows and concluded that daily rumination time was only associated with age but not with the breed or genetic merit of the cow. Pereira and Heins (2019) also looked into production systems comparing cows reared in organic grazing environment and low input conventional environment, and observed that cows in organic management system have greater rumination time except during summer months (June, July, and August).

Rumination is considered to be an indicator of feeding and lying behavior of cows. Schirmann et al. (2012) studied 42 Holstein dairy cows for their feeding and rumination behavior in the early dry period and observed that cows spent more time ruminating after periods of high feed intakes. RT is also found to be affected by diet. Diets containing 11.7% NDF resulted in 12.7% less rumination time than diets with 14.1% NDF; with 23.5% increase in RT per kilogram of roughage ingested (Adin et al. 2009). Beauchemin and Yang (2005) also support the fact that RT linearly increases as dietary physically effective NDF. Furthermore, total RT was increased when saturated fat was supplemented.

Bender et al. (2014) evaluated daily variation in body weight, milk production, and rumination activity in dairy cows and observed rumination to average 461.1 min/day, with a standard deviation of 6.1 min among days within a pen, 128.0 min among individual cows within a pen, and 43.6 min among days within individual cows. DeVries and Chevaux (2014) supplemented dairy cows with live yeast (1 × 10^10^ cfu/head per day) and found that the supplemented cows ruminated longer (570.3 vs 544.9 min/d; SE ± 0.04 min). The authors attributed this increase in rumination time to frequent smaller meals in closer time together. In another study from the same group, DeVries (2009) indicated that high grain intake leads to acute risk of rumen acidosis that leads to lower rumination than cows in the low risk group (high risk cow ruminating 1 h less than low risk cow per day). The authors attribute this difference to the variation in amount of forage content in the two diets, with low forage group leading to reduced rumination time.

Yang and Beauchemin (2007) suggested that physically effective fiber in the dairy cattle diet increased rumination time, indicating that forage to concentrate ratio and forage particle length has direct relation with total rumination time as well as rumination time per kg of dry matter. Schmitz et al. (2019) identified that daily rumination time was reduced with high energy concentrate diet (453 vs 457 min/d; *p* = 0.001). On a similar study, Salfer et al. (2018) identified that high fiber-low starch diets caused greater daily rumination time. Rumination was observed to be in a 24 h rhythm with amplitude of the rumination being reduced in low starch diets and diets containing saturated fatty acids or a mixture of saturated and unsaturated fatty acids. Decreasing NDF concentration also decreased the amplitude of the daily rumination rhythm when conventional corn silage was fed. Salfer et al.(2018) also suggested that fat supplementation also affected the amplitude of daily rumination rhythms. Supplementation with SFA or a mixture of SFA and UFA increased the amplitude of the daily rhythms. Alternatively, supplementation with UFA alone had no effect. In this study, the daily pattern of rumination was affected by NDF and starch concentrations, with low-NDF, high-starch diets causing the greater reduction in rumination during the overnight period than during mid-day. Overall, high concentrations of physically effective fiber increase rumination and salivation, thus increasing rumen pH (Beauchemin and Yang 2005).

Sjostrom et al. (2016) concluded that the daily rumination time was greater during September (402 min/d) compared to July (361 min/d). Clément et al. (2014) developed dry matter intakes and stated that the rumination time estimate has a significant role in the DMI prediction model. Heinrichs et al. (2021) evaluated the rumination time in the TMR-restricted cows and identified that limit feeding cows with or without hay ruminated for a large amount of time during the hours of TMR restriction.

This narrated review seeks to understand different areas where rumination time could be utilized in monitoring dairy cattle, including calving, estrus detection, heat stressed conditions, and to detect diseases and transition cow disorders. A systematic literature search was conducted to identify peer reviewed publications in English language that discuss rumination monitoring in dairy cattle. Electronic databases were assessed through the server of the Texas A&M University including: PubMed (Medline, 1940–2021), Web of Science (Thompson Reuters, 1945–2021), Science Direct (Elsevier, 1927–2021), and Scopus (Elsevier, 1960–2021). The search strategy included the following keywords: (‘dairy cow*’ OR ‘dairy cattle’ OR ‘lactating cow*’ OR ‘lactating dairy cow*’ OR ‘periparturient cow*’) AND (disease* OR ‘metabolic disease’ OR ‘metabolic disorder’ OR SCK OR ketosis OR ‘transition period’) AND (‘ruminat* time’ OR ruminat* OR ‘ruminat behavio*’). Additionally, the search was supplemented with physiological and behavioral term (Rumination, chewing the cud) and commercial name (e.g. SCR, CowManager). Research papers that contain information pertinent to rumination in dairy cattle were included in this study. The research was carried out between February and March 2021 and updated in May 2021.

## 2 Rumination time and onset of calving

Cows spend relatively less time ruminating when parturition approaches. There is a distinct rumination behavior during the first week after calving; RT dramatically decreases at the day of calving and recovers quickly in the following week (Bar 2010; Schirmann et al. 2013; Paudyal et al. 2016). In a study by Buchel and Sundrum (2014), 15 out of 17 (88%) of the dairy cows analyzed showed reduction in RT by a mean of 27% (25.6 min/6 h) during the last 6 h of calving. Similarly, Pahl et al. (2014) studied the rumen activity of dairy cows 24 h before and after calving in a total of 17 cows and found that the RT decreased in the last 4 h antepartum and in the first 8 h postpartum. Borchers et al. (2014) suggested that using activity measurement and RT was useful in predicting impending calving without any other new technology or parameter being used. In another study by Calamari et al. (2014), the average RT before calving was 479 min/d, which reached a minimum value at calving (i.e. 30% of RT before calving). The relationship demonstrated between the RT and calving time constitute a new opportunity for predicting the timing of calving (Schirmann et al. 2013; Buchel and Sundrum 2014). Mammi et al. (2021) observed lower postcalving RT in cows with difficult calving suggesting longer impact of calving stress on the daily RT.

## 3 Rumination time and detection of estrus

Reith and Hoy (2012) studied 265 estrus events from 224 dairy cows with artificial insemination leading to conception. In the estrous cows RT was significantly reduced. The average decrease in RT was 17% (74 min/d) ranging from −71 to +16% among animals and between 14 (60 min/d) and 24% (94 min/d) among herds with the decrease in RT more pronounced in primiparous than in multiparous cows.

Reith et al. (2014) furthered research to analyze the activity and RT in the peri-estrus period and confirmed that cows in estrus spent significantly less time ruminating and the activity level was significantly increased during the period.

Pahl et al. (2015) evaluated the changes in RT of 62 dairy cows around estrus. The study found that the RT was significantly decreased on d −1 and 0 with the RT of 77 min (day −1) and 75 min (day 0) less than on the reference day. The extent to which rumination time decreased did not differ among primiparous and multiparous cows in this study. A more recent study (Minegishi et al. 2019) observed decreased rumination time in 82% of the estrus events. However, the sensitivity and specificity of estrus detection decreased when the cows had access to pasture. This suggests a need to establish an algorithm that considers altered activity and rumination in cattle during grazing.

More interestingly, another study (Schweinzer et al. 2020) observed that cows coming to heat naturally demonstrated a clear drop in rumination time, whereas cows with induced estrus only showed minor changes in behavioral patterns during estrus. They also concluded that cows under estrus synchronization protocol (e.g. Ovsynch) showed minor changes in rumination patterns.

## 4 Rumination time and detection of diseases

Feed intake, feeding, and RT are considered as important parameters for the identification of suboptimal feeding conditions, and can be used to indicate possible health disorders (Buchel 2013). RT is associated with the metabolic condition and disease state of dairy cattle around parturition. Therefore, rumination monitoring may be helpful to quickly obtain information on the health status of animals in a critical period like the transition phase (Siivonen et al. 2011; Soriani et al. 2012; Mammi et al. 2021). Monitoring RT around calving and in particular during the first week of lactation has been proposed to be an effective means to identify the cows that are at a greater risk of developing disease in early lactation (Calamari et al. 2014). The early detection of clinical and subclinical disease through rumination monitoring allows farmers to initiate the treatment that could reduce the costs associated with the treatment of chronic cases and more severe production loss (Carraway et al. 2013). Furthermore, the time required for normalization of eating and rumination behavior in a sick animal has prognostic value and may be taken as a parameter of the effectiveness of the applied treatment (Braun et al. 2013).

Decreased rumination time has been associated with the stress, anxiety and diseases (Welch et al. 1970; Hansen et al. 2003). Rumination time before calving may be an indicator of health during early lactation. Soriani et al. (2012) monitored the rumination pattern during the transition period to investigate its relationships with metabolic conditions, milk yield and health status and reported that the rumination time was positively correlated with milk yield (*r* = 0.36). Cows with reduced rumination time before calving maintained reduced RT after calving and suffered a greater frequency of disease than cows with greater RT in late pregnancy. Cows with mild inflammatory conditions or without health disorders during parturition showed a greater average rumination time (>520 min/d) during the10 d after parturition. On the other hand, decreased RT (<450 min/d) during the first few days of lactation was observed in cows with subclinical diseases or health disorders (Soriani et al. 2012). Calamari et al. (2014) observed that more than 90% of the cows that had low RT before parturition had clinical illness in early lactation; whereas only 42% of the high ruminating cows had clinical illness. Paudyal et al. (2018) used two indices that could satisfactorily identify different health disorders using animal level and pen level comparisons. The cow level index compared daily rumination with the 7 day rolling average of the same cow and the pen level index compared daily rumination time with the average of the cows in the same herd. This approach utilized deviations in rumination, which accounts for variations in rumination time between the cows and daily variation within the same cow.

Cows affected by clinical mastitis demonstrated a reduction of RT and a change in its variability some days before antimicrobial treatment (Soriani et al. 2012). Chapinal et al. (2014) studied the effect of flunixin meglumine on rumination in dairy cows with endotoxin-induced mastitis. Cows challenged with intramammary infusion of *Escherichia coli* lipopolysaccharide (LPS) and not treated with the drug (control group) ruminated less than treated cows 5–8 h and 11–12 h after LPS infusion. Thus, experimentally induced mastitis has an effect of reducing the rumination time. In another similar study, Fitzpatrick et al. (2013) studied the effect of meloxicam on rumination time in dairy cows with endotoxin induced mastitis. Cows spent significantly less time ruminating in the hours after LPS infusion and compensated with more time ruminating later in the day. Thus, altered RT can be related to mastitis.

Van Hertem et al. (2013) investigated the utility of continuous monitoring of milk production and rumination activity for lameness detection. The investigators found that the highest correlation of lameness with a rumination variable was on day 6 before diagnosis for the nighttime RT and the correlation coefficient was 0.21 (*p* = 0.007) for RT-related behaviors. However, other studies suggested minimal changes. Weigele et al. (2018) suggested that moderate lameness leads to changes in some behavioral parameters but rumination behavior was not found to be significantly different. King et al (2017) also concluded that there was no difference in daily rumination or activity between lame and nonlame cows or in night:day rumination time. This concept was supported by another research (Thorup et al. 2016) which also concluded that lameness in dairy cattle affects cow feeding but not rumination behavior obtained from sensor systems.

### 4.1 Rumination time and transition cattle disorders

Dairy cows are most susceptible to become ill during the transition period (i.e. 3 wk before to 3 wk after calving) (Stevenson et al. 2020). The transition period in cattle is typically characterized by a decline in feed intake beginning 3 wk before calving, depression of certain immune functions both before and after calving, a negative energy balance, and decline in serum calcium and glucose at the onset of lactation. Animals after calving sacrifice immune function for the sake of maintaining lactation (von Keyserlingk et al. 2009). Most postpartum diseases are complex and they have multiple causation. For instance, many infectious diseases diagnosed during transition occur as secondary illnesses to metabolic diseases such as ketosis or hypocalcemia (von Keyserlingk et al. 2009). Transition period cows are also subjected to regrouping as they move into pre-calving groups and then into the lactating herd, and there is evidence of decreased rumination in pre-partum cows that were moved to a new social group (Schirmann et al. 2011). Furthermore, early postpartum rumination time indicating low stress calving was associated with peak milk production in over the entire lactation. Peiter et al. (2021) observed that for each 100 min increase in RT, lactation peak daily milk was increased by 2.2 kg in multiparous Holstein cows. In a similar recent study, Stevenson et al. (2020) suggested that healthy cows spent less time being inactive during both pre-partum and post-partum periods compared with diseased cows and had greater postpartum rumination times than diseased cows. More interestingly, rumination in healthy cows increased rapidly to a peak on 8 days in milk and plateaued off, whereas the peak in rumination in diseased cows was delayed to 15 days before leveling off.

There is evidence that knowledge of rumination behavior can help identify transition dairy cows at risk for metritis, subclinical ketosis, and lameness. This information could also be used to guide the development of management practices that can help detect diseases early and help to prevent disease by addressing management challenges during the transition period (von Keyserlingk et al. 2009).

Cows that experienced metritis in the transition period demonstrated different feeding behavior and spent less time feeding during both pre- and post-calving periods (von Keyserlingk et al. 2009). Cows developing metritis also ate less than healthy cows in the pre-partum period (Huzzey et al. in von Keyserlingk et al. 2009). When daily rumination time was used to detect the cows with severe metritis it was possible to define thresholds of rumination time during the first six days of lactation to detect the cows with health disorders (Soriani et al. 2013a). These authors observed highly significant differences in rumination behavior between cows affected by severe metritis and healthy cows during the first week of lactation.

Goldhawk et al. (2013) observed that cows with low pre-partum intakes were also more at risk for subclinical ketosis after calving. Cows that later developed ketosis ate less and spent less time eating. Thus, suggesting that the feeding behavior of cattle during the transition can be used to predict metabolic disease. The presence of severe ketosis or mild metritis or retained placenta affected the RT during the 6th days in milk (DIM). Severe ketosis and retained placenta also affected the rumination time on the 5th DIM and cows affected by retained placenta demonstrated reduction of daily RT during the 2nd DIM (Soriani et al. 2013a).

Schirmann et al. (2013) also studied the rumination behavior before calving and its association with metritis and subclinical ketosis postpartum. As compared to healthy cows, cows with subclinical ketosis or metritis and subclinical ketosis together, spent less time ruminating in the pre-partum period. However, there was no difference between healthy and any of sick groups in time spent ruminating after calving. Thus, RT information before calving show promising results in identifying cows at risk for metritis and subclinical ketosis after calving. Recent research by Cocco et al. (2021) also identified rumination time as a good predictor of subclinical ketosis in both pre-partum and post-partum dairy cows. This research concluded milk production and parity affects rumination time and ration characteristic like crude protein, net energy and NDF influenced the rumination time in dairy cattle.

Liboreiro et al. (2014) studied peri-partum health events and RT and concluded that cows with retained placenta had reduced cud chewing time. These investigators identified an interaction effect of subclinical hypocalcemia and days relative to calving on RT. Similarly, another interaction of ketosis and days relative to calving was observed on RT. Serum concentrations of calcium and beta hydroxybutyrate were also related with RT. In a similar study, Sterrett et al. (2014) observed no differences in RT between subclinical hypocalcemia (HYC) and non-HYC cattle and also in subclinical ketosis and non-subclinical ketosis cattle. Bar and Solomon (2010) compared average daily RT of milking cows on days without any event to days with either nutritional changes, mastitis, calving or estrus and observed a clear significant decrease in RT on days with these events. This supports the usefulness of RT to track potential individual cow health problems, deviation from normal behavior and to monitor the effects of intentional or accidental nutritional changes in herd. A recent study by Goff et al. (2020) observed that cows with normal calcium levels spent more time ruminating after calving than subclinical hypocalcemia or cows with clinical signs of milk fever. They also observed that cows fed DCAD diet during dry period ruminated 86 min longer than control cows which emphasizes that dry cow management has long-term impact in the post-partum cows.

## 5 Rumination time and heat stress

Rumination can also be an asset to determine the heat stress level of dairy cattle. Acatincai et al. (2009) concluded that when temperature exceeds the upper limit of the thermal comfort of a particular breed, rumination process is severely affected. Temperatures beyond 27–28 °C reduce the overall rumination process, including both frequency and duration of this activity. Soriani et al. (2013b) observed that in dairy cows suffering mild to moderate heat stress there was a negative relationship between daily maximum temperature-humidity index (THI) and RT (*r* = −0.32), with a decrease of 2.2 min of RT for every daily maximum THI unit over THI of 76. Rumination time was negatively associated with breathing rate and positively related to milk yield (Soriani et al. 2013b). In agreement with previous studies, Moretti et al (2017) identified a Pearson’s correlation between RT and THI showed a significant unfavorable correlation (−0.22, *p* < 0.001). Müschner-Siemens et al. (2020) observed that RT is affected by several individual cow factors even in moderate climate indicating that the parameter could be used to evaluate effect of climate change in dairy cows. According to another research (Ji et al. 2020), when the thresholds temperature exceeds, a 1 °C increase of daily mean temperature decreased rumination time by 5.12 min per day and reduced rumination efficiency by 0.07 kg per cow per hour. In another study, total rumination time, day time rumination, and nighttime rumination time were all decreased with high THI in early, mid, and late lactation stages of dairy cattle (Abeni and Galli 2017). More interestingly, heat stress reduced the rumination time by 22.9% even in dry dairy cows leading to impaired the ruminal degradability of the consumed feed (Maia et al. 2020).

## 6 Conclusions

Monitoring rumination time provides opportunities for the early detection of health disorders and to optimize reproduction in the dairy cows. Different factors account for the variation in rumination time, and the decreased rumination time provides opportunities be used as an indicator of the health status in dairy cattle. Although most of the studies support use of the system in disease detection, further research is warranted to evaluate different approaches to refine the detection algorithm and to combine rumination time with other production and behavioral variables to obtain a system with high sensitivity and specificity of disease detection.
